# The management of microbial keratitis within Uganda’s primary health system: a situational analysis

**DOI:** 10.12688/wellcomeopenres.15463.1

**Published:** 2019-09-24

**Authors:** Simon Arunga, Naome Kyomugasho, Teddy Kwaga, John Onyango, Astrid Leck, David Macleod, Victor Hu, Matthew Burton

**Affiliations:** 1 Ophthalmology, Mbarara University of Science and Technology, Mbarara, Uganda; 2 International Centre for Eye Health, Clinical Research Department, London School of Hygiene & Tropical Medicine, London, WC1E 7HT, UK; 3 Ruharo Eye Centre, Ruharo Mission Hospital, Mbara, Uganda; 4 Tropical Epidemiology Group, London School of Hygiene & Tropical Medicine, London, WC1E 7HT, UK

**Keywords:** Microbial Keratitis, Bacterial Keratitis, Fungal Keratitis, Blindness, Uganda Health System

## Abstract

**Background**: Microbial keratitis (MK) frequently leads to sight-loss, especially when the infection is severe and/or appropriate treatment is delayed. The primary health system as an entry point plays a central role in facilitating and directing patient access to appropriate care. The purpose of this study was to describe the capacity of primary health centres in Uganda in managing MK.

**Methods**: We carried out a rigorous assessment of primary health centres and mid-cadre training schools in South Western Uganda. Through interviews, checklists and a picture quiz, we assessed capacity and knowledge of MK management. In addition, we interviewed the heads of all the mid-cadre training schools to determine the level of eye health training provided in their curricula.

**Results**: In total, 163 health facilities and 16 training schools were enrolled. Of the health facilities, only 6% had an Ophthalmic Clinical Officer. Only 12% of the health workers could make a diagnosis of MK based on the clinical signs in the picture quiz. Although 35% of the facilities had a microscope, none reported doing corneal scraping. None of the facilities had a stock of the recommended first line treatment options for MK (ciprofloxacin and natamycin eye drops). Among the training schools, 15/16 had an eye health component in the curriculum. However, the majority (56%) of tutors had no formal expertise in eye health. In 14/16 schools, students spent an average of two weeks in an eye unit.

**Conclusions**: Knowledge among health workers and capacity of health facilities in diagnosis and management of MK was low. Training for eye health within mid-cadre training schools was inadequate. More is needed to close these gaps in training and capacity.

## Introduction

Microbial keratitis (MK) can be caused by a range of pathogens including, bacteria, viruses, protozoa, and fungi. It is characterized by acute or sub-acute onset of pain, conjunctival hyperaemia and corneal ulceration with a stromal inflammatory cell infiltrate ^1^. MK frequently leads to sight-loss from dense corneal scarring, or even loss of the eye, especially when the infection is severe and/or appropriate treatment is delayed.

MK has been described as a ‘silent epidemic’, which leads to substantial morbidity, related to blindness, pain and stigma ^2^. It is the leading cause of unilateral blindness after cataract in tropical regions, estimated at 2 million cases of monocular blindness per year ^3^. In 2017, 1.3 million individuals were bilaterally blind from corneal opacity globally (excluding trachoma and vitamin A deficiency), accounting for 3.2% of binocular blindness ^4^. In Sub-Saharan Africa (SSA), MK is an important cause of binocular blindness and is responsible for about 15% of monocular blindness ^5, 6^.

A good outcome depends on early appropriate treatment, supported by correct identification of the causative organism, and careful follow-up ^7, 8^. In low and middle-income countries (LMICs), these resources are not readily available and outcomes tend to be poor ^9^. It is important for patients to present early when the infection can be more easily controlled; for instance, studies in Burma and Bhutan showed that if people responded within the first 24–48 hours to a corneal abrasion by applying antibacterial or antifungal medication, there would be 100% recovery without any sequalae ^10, 11^. Once a corneal ulcer is fully established, there is little that can be done to change its course ^12^.

The primary health system plays a central role in facilitating and directing patient access to appropriate care. A retrospective study from Tanzania found that having visited a health facility was, paradoxically, a risk factor for severe presentation among patients with MK ^9, 13^. Our previous work found that although the majority of patients lived within 5km of their nearest primary health centre (PHC) and presented early to those facilities, they ended up presenting very late to eye hospitals and with severe infection when little could be done ^14^. There were several missed opportunities at this entry level into the health care system to diagnose, manage and or promptly refer these patients.

The purpose of this study was to conduct a situation analysis of knowledge, practise and capacity of the PHCs in management of MK and to determine the level of training offered on eye health to mid-level cadres in Uganda.

## Methods

### Ethical statement

This study followed the tenets of the Declaration of Helsinki. It was approved by the London School of Hygiene & Tropical Medicine Ethics Committee (Ref 10647), Mbarara University Research Ethics Committee (Ref 10/04–16) and Uganda National Council for Science and Technology (Ref HS-2303). Permission was sought from the District Health Offices to approach the facilities. Then, written informed consent was obtained from the facility and school heads before enrolment to participate in the study and to allow the research assistants collect information about their facilities. Written informed consent was also obtained from each facility head, school head and health worker for their personal participation in the research.

### Study design and setting

The sampling frame for this study was defined by the catchment area for a related cohort study that prospectively enrolled all patients presenting with MK to the main tertiary referral centres for South Western Uganda (Ruharo Eye Centre [REC] and Mbarara University and Referral Hospital Eye Centre [MURHEC]) from December 2016 to March 2018. The districts from which these patients came from were identified by tallying the district data of MK patients and seeing which districts had large numbers of MK patients. These were then pooled into regions depending on their geographical distribution and distance from the eye hospital. One district was then randomly selected from each pool.

Uganda’s Health System is a tier-based system divided into seven levels with the lowest point of care being at the village level. However, physically, a Health Centre II (HC II) is the lowest unit and is located at a parish level. These units have different staffing and capacity in terms of service provision. Patients are referred along the tier system depending on the complexity of their condition. Special Clinics are facilities that provide specialised services only, such as HIV treatment services. There is a total of 408 HC IIs, 152 HC IIIs, 43 HC IVs and 12 hospitals within the 20 districts in South Western Uganda. We randomly selected six districts, stratified by geographical distribution and accessibility from the eye hospital in Mbarara. All the health facilities within the sampled districts were enrolled. In addition, all the mid-cadre training schools (nursing and clinical officers) within South Western Uganda were enrolled.

### Data collection

***Quantitative interviews***. Research assistants administered questionnaires (File 1, *Extended data*) ^15^ to facility heads and/or health workers who treat eye patients to ascertain the level of knowledge, routine practices and capacity. One health worker was interviewed per facility. Heads of mid-level cadre training schools were interviewed using questionnaires to ascertain the amount of eye health training provided (File 2, *Extended data*) ^15^.

***Checklists***. Data were collected from the health centres we visited about infrastructure, equipment and supplies relevant to managing MK. The research assistants collected this information from the facility health workers and directly tallied patient register entries to count the number of eye patients in general and MK cases that visited that unit, as well as diagnosis and treatment. A copy of the data collection tool has been provided as *Extended data* (File 1).

**Figure 1.**
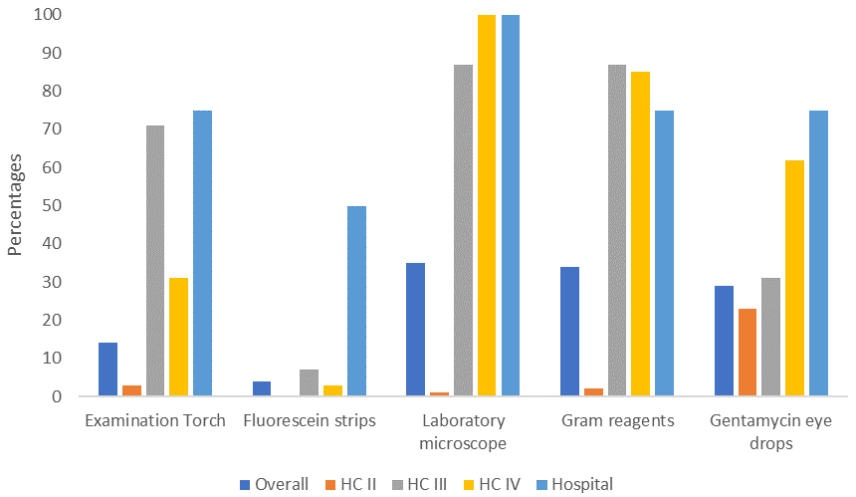
Basic inventory of health facilities for detecting and managing microbial keratitis (n=163).

***Picture quiz***. A photograph of an eye with classic signs of MK was given to the health workers (one health worker per facility) to test their knowledge of clinical signs and ability to diagnose MK ( Figure 1, File 3, *Extended data*) ^15^. The quiz was a section of the general questionnaire (File 1, *Extended data*) ^15^ that was administered. The health workers were shown a coloured picture of an infected eye that had clinical signs of MK. They were then asked to identify the clinical signs and suggest a diagnosis and management plan.

### Analysis

Data were analysed in STATA 14 (StataCorp). Summary tables were used to describe knowledge, inventory, proportion of eye patients seen. This was stratified by level of the health centres. The same analysis was used for training schools to describe training key training areas in the identification and management of MK.

## Results

A total of 163 health facilities were enrolled from six districts in South Western Uganda (101 HC IIs, 45 HC IIIs, 13 HC IVs and four district hospitals). These were from Kamwengye district (27), Kisoro district (31), Ntungamo district (40), Sheema district (26), Lyantonde district (16) and Ssembabule district (23). Table 1 shows the baseline characteristics of the enrolled health facilities. Only five facilities had an Ophthalmic Clinical Officer (OCO): these included four hospitals and one HC IV ^15^. Most of the facilities (59%) were headed by an enrolled nurse cadre. Most (74%) facilities had less than 50% of the expected staffing levels. The proportion of patients seen at these facilities who presented with eye problems was 2–8%.

**Table 1.** Baseline characteristics of the enrolled health facilities (n=163).

Variable	Count	(%)
**Level of the health centres (HCs)**		
HC II	101	(62%)
HC III	45	(28%)
HC IV	13	(8%)
Hospital	4	(2%)
**Distance to nearest referral centre**		
0–5km	27	(17%)
6–10km	72	(44%)
11–20km	43	(26%)
>20km	21	(13%)
**In charge cadre type**		
Enrolled nurse	96	(59%)
Clinical officer	39	(24%)
Medical officer	18	(11%)
Other	10	(6%)
**Staffing levels**		
0–25%	42	(26%)
26–50%	79	(48%)
51–75%	32	(20%)
>75%	10	(6%)
**Patient registry data**		
Variable	**Median**	**(IQR)**
**Number of patients seen in last three months**		
HC II	3421	(1350-4782)
HC III	3187	(2160-4213)
HC IV	4625	(2296-6251)
Hospital	8301	(6181-14146)
**Proportion of eye patients in last three** **months in percentage (range)**		
HC II	1.0%	(0.5-2)
HC III	2.5%	(1-5)
HC IV	2.0%	(1-6)
Hospital	8.0%	(3-14)

161/163 had an all-weather road access and 158/163 had a phone communication at the facility. 136/163 had a power supply.

Figure 1 illustrates, by level of the health facility, the availability of basic diagnostic tools and treatment for MK (Supplementary Table 2, File 3, *Extended data*) ^15^. Overall, 14% of the facilities had an examination torch, 4% had fluorescein strips for corneal staining, 35% had laboratory microscopes and Gram staining facilities. When we looked at eyedrops relevant to MK, 29% had gentamycin eye drops available. However, none of the facilities had ciprofloxacin or natamycin eye drops. There was a systematic difference in this capacity across the level of the health centres, with higher facilities being better equipped.

**Figure 2.**
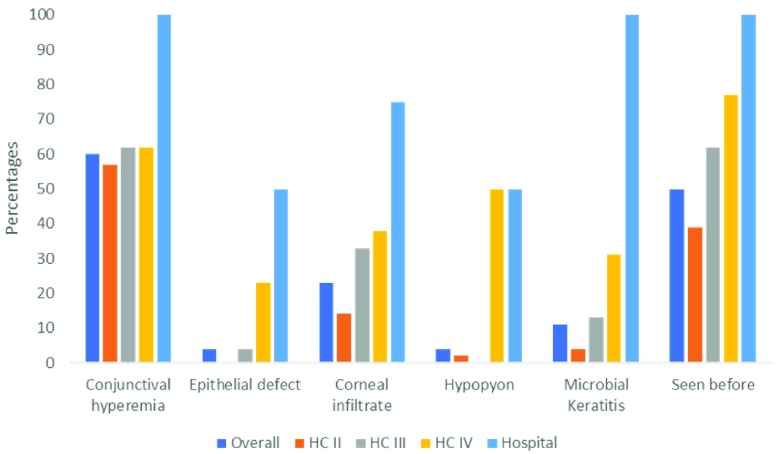
Knowledge of clinical signs of microbial keratitis among primary health workers (n=163).

Knowledge of clinical signs of MK was tested using a picture quiz of a patient’s eye with MK ( Figure 1, File 3, *Extended data*) ^15^. The results are presented in Supplementary Table 3 (File 3, *Extended data*) ^15^. Overall, 60% of the health workers identified a red eye. However, only 4% identified an epithelial defect, 23% a corneal infiltrate and 4% recognised a hypopyon ( Figure 2). On being asked what the most likely diagnosis was, only 12% of the health workers could correctly identify it as MK or eye infection. There was a systematic difference of the knowledge by level of the facility.

**Figure 3.**
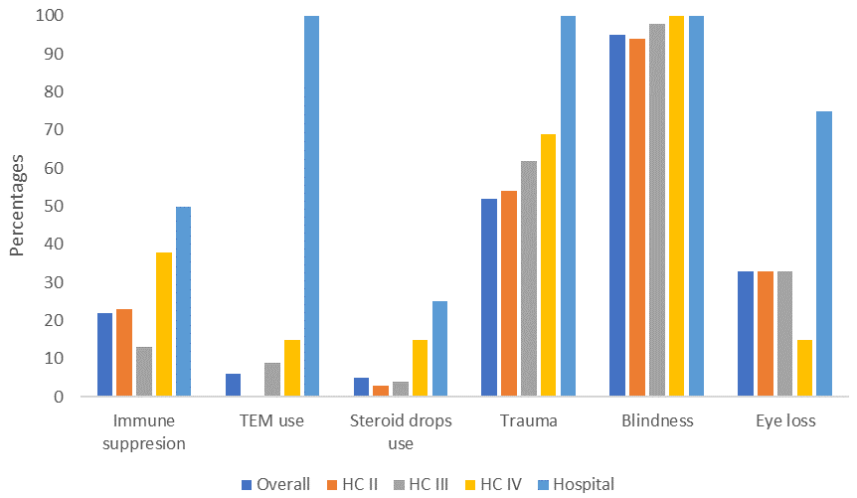
Knowledge of risk factors and complications of microbial keratitis among primary health workers (n=163).

Figure 3 shows knowledge of risk factors and complications of MK among the primary health workers (Supplementary Table 3, File 3, *Extended data*) ^15^. Overall, 22% identified immune suppression, 5% identified traditional eye medicine (TEM), 5% identified steroid eye drops, 50% identified trauma. When asked to name complications of MK, 95% of the health workers reported blindness. However, only 33% mentioned eye loss. On stratifying by level, there was not much difference in this knowledge across the different level facilities.

**Figure 4.**
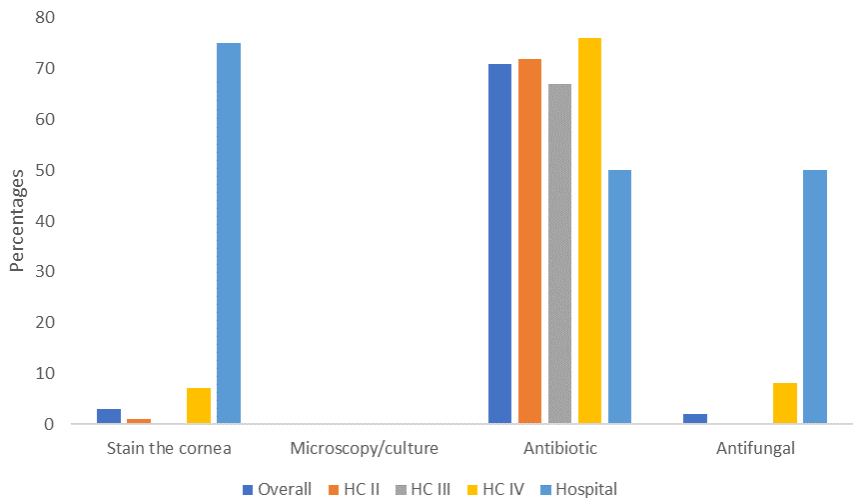
Knowledge on management of microbial keratitis among primary health workers (n=163).

Figure 4 shows the knowledge of management of MK among the primary health workers (Supplementary Table 3, File 3, *Extended data*) ^15^. Overall, only 3% knew about staining of the cornea with fluorescein to examine for epithelial defects in the diagnosis of MK. None of the cadres, including the OCOs, showed knowledge of the role of microscopy/culture in management of MK. Antibiotic as a choice of treatment was reported in majority of the cadres across all levels. However, antifungal was reported by only the OCOs. This is not surprising as antifungal eye drops are not commonly available.

There are 22 mid-cadre training schools in South Western Uganda. We included 16 in this study. The rest declined to participate. The findings are summarised in Table 2. Overall, 15/16 schools had an eye health component in their curriculum, 14/16 schools provided eye clinic rotations for their students. However, the majority (56%) of the trainers/tutors had never had any formal training in ophthalmology. Although training of practical skills during eye ward attachments was excellent for basic areas, critical skills for management of MK and other emergency conditions such as staining, removal of a foreign body, using loupes to examine eyes were not being taught.

**Table 2.** Assessment of capacity of eye health training in mid-cadre schools (n=16).

	Overall n=16		Certificate level, n=6		Diploma level, n=10	
**Variable**	**Count**	**(%)**	**Count**	**(%)**	**Count**	**(%)**
**Presence of an eye health curriculum**						
Yes	15	(94%)	6	(100%)	9	(90%)
Major topics covered in the curriculum (if Yes) *						
Anatomy	14	(94%)	6	(100%)	8	(89%)
Blinding conditions	12	(80%)	5	(83%)	7	(78%)
Eye infections	15	(100%)	6	(100%)	9	(100%)
Pharmacology	13	(87%)	4	(67%)	9	(100%)
**Ophthalmology training level of the eye health tutor**						
None	9	(56%)	5	(83%)	4	(40%)
Diploma	7	(44%)	1	(17%)	6	(60%)
**Does hospital attachment include eye ward? (if Yes; median 2 weeks** **IQR 1-2, total range 1–6 weeks)**						
Yes	14	(88%)	5	(83%)	9	(90%)
**Practical skills learnt in the eye ward** Ɨ						
Taking ocular history	14	(100%)	5	(100%)	9	(100%)
Measuring visual acuity	14	(100%)	5	(100%)	9	(100%)
Instilling eye drops	14	(100%)	5	(100%)	9	(100%)
Eye exam with a torch	14	(100%)	5	(100%)	9	(100%)
Corneal staining	5	(36%)	2	(40%)	3	(33%)
Eye exam with loupes	1	(7%)	1	(20%)	0	(0%)
Ophthalmoscopy	4	(29%)	1	(20%)	3	(33%)
Foreign body removal	1	(7%)	1	(20%)	0	(0%)

*Only schools which had an eye health curriculum were analysed (n=15). Ɨ n=14, two schools did not have students rotate in an eye ward. Certificate level means certificate in nursing, diploma level means diploma in nursing or clinical medicine.

## Discussion

This study aimed to assess the capacity of the PHCs in managing MK. We found that there was a lack of essential personnel for eyecare at the PHCs. Human Resources for Health (HRH) are a huge problem for many places in SSA. This is particularly an issue for Human Recourses for Eye Health (HREH), with many countries in SSA failing to achieve the Vision 2020 staffing targets ^16, 17^. In our study, the majority of the facilities were substantially understaffed, with more than half having severe staffing shortages. According to the Uganda Ministry of Health staffing norms, each HC IV and above are supposed to have an Ophthalmic Clinical Officer (OCO). However, in our study, only 1/13 HC IVs had an OCO. This means that majority of the eye patients are seen by general health workers who have poor knowledge on eye care ^18, 19^.

One study from Tanzania reported limited knowledge and productivity for eye care among primary health workers. In that study, the primary workers were found wanting in basic skills including measuring of visual acuity ^19^. Another study that looked at knowledge of primary eye care among 343 general health workers from Kenya, Tanzania and Malawi also found low knowledge levels for common conditions; only 8.2% of the workers could correctly measure visual acuity ^18^.

Therefore, it is not unexpected that we found that the majority of health workers could not correctly identify the signs of microbial keratitis, one of the more common ophthalmic emergencies, and provide a satisfactory management plan. This limited knowledge will most likely compromise patient care and lead to poor outcomes. For example, a very small proportion of the health workers identified use of steroids as a risk factor for MK, which might imply that they would consider treating potential MK patients with steroids, possibly making it worse. Our experience in treating MK patients presenting to us is that the majority have used a combination of eye drops, most containing a steroid.

The health facilities were poorly resourced in basic items for eye care such as examination torches, fluorescein for corneal staining and drugs for treatment. We did not find a single health facility that had a stock of ciprofloxacin and/or natamycin eyedrops, which are the recommended first line agents for bacterial and fungal keratitis, respectively ^20– 22^.

Despite these discouraging findings, there were some positive findings that could be utilised to improve care. Most health workers were able to identify conjunctival hyperaemia (red eye) on the picture quiz. This would be a good starting point for a training pack on managing emergency eye conditions. We recently published two articles targeting primary health workers on how to identify MK and how to locally make fluorescein for corneal staining, and a more recent version of a WHO primary eye care training manual for Africa has been made available ^23– 25^. We hope to use these resources for training general health workers on triage and emergency management of acute ocular conditions.

In addition, we found that a modest number of facilities had microscopes and laboratory personnel. With simple training and simple tools such as magnifying spectacles/loupes, the health workers could be in a good position to do corneal scraping and microscopy to differentiate between bacterial and fungal keratitis. Laboratory diagnosis for MK is still the most reliable method since some clinical presentations are equivocal and even corneal specialists may make the wrong clinical diagnosis ^26^. However, corneal scraping requires a certain level of competence and would be feasible only at a limited number of centres which have OCOs. The other health cadres not suitable to do corneal scraping can be equipped to identify MK and provide early referral. Simple tools such as corneal staining with fluorescein have been used in large studies in Bhutan and Burma among minimally trained health workers to facilitate early detection, treatment and referral of MK ^10, 11^. In these trials, village health workers were able to stain the cornea with fluorescein and examine for corneal abrasions using a blue light torch.

Some facilities also had a stock of gentamycin 0.3% eye drops available. This can be locally fortified to make a more potent alternative of 1.5% gentamycin, which is quite effective for managing some forms of bacterial keratitis ^27, 28^. In Uganda, both gentamycin eyedrops and parenteral gentamycin vails (that can be used to fortify the eye drops) are available and supplied to the facilities through the free National Medical Stores. However, gentamycin is limited to Gram negative bacteria and does not cover Gram positive bacteria and fungi. Quinolones such as ciprofloxacin have a broader coverage. These, together with natamycin for fungal keratitis, are on the WHO essential medicines list and should be availed to lower facilties ^20– 22^.

In this study, we also assessed mid-cadre training schools to assess the scope of training on eye care. Overall, we found that most schools had an eye health component in their curriculum and students were getting some time (although little) to have hands on training rotations in an eye unit.

However, the levels of ophthalmology training among the trainers were low. The majority of the trainers, especially in certificate-level training schools, had never received any structured courses in ophthalmology except as part of the short rotations during their medical training. Therefore, this casts doubt on the quality of training they can offer on eye health.

The training of practical skills during eye ward attachments was reported to be excellent for basic areas such as taking ocular history, measuring visual acuity, eye examination with a torch and instilling eye drops. However, critical skills for management of MK and other emergency conditions such as staining, removal of a foreign body and using loupes to examine eyes were not being taught.

## Limitations and strengths

This study enrolled a large sample of health facilities and used multiple sources of data collection. The findings from the training schools were reported by the school heads and therefore might have a degree of bias. We were not able to conduct an exit assessment among the students to verify if these learnings reported by the school heads were accurate.

## Conclusion

The findings from this study draw attention to the very limited quality of eye care at the PHC, but it also points to several opportunities that could be utilised to improve this. The knowledge among health workers and capacity of health facilities in diagnosis and management of MK was low. Training for eye health among mid-cadre training schools was inadequate. More is needed to strengthen these gaps in training and capacity.

## Data availability

### Underlying data

Harvard Dataverse: The management of microbial keratitis within Uganda’s primary health system: a situational analysis. https://doi.org/10.7910/DVN/MSLAOS ^15^.

This project contains the following underlying data

- Health Facilities data.xlsx (quantitative underlying data)
- Training schools dataset.xlsx (quantitative underlying data)

### Extended data

Harvard Dataverse: The management of microbial keratitis within Uganda’s primary health system: a situational analysis. https://doi.org/10.7910/DVN/MSLAOS ^15^.

- 7.MAES HEALTH CENTRE data collection forms-v3-31oct2018.docx (File 1: a copy of the data collection forms used on the health facilities which include sections on the checklist and picture quiz)
- 7.MAES SCHOOLS data collection forms-v1-25FEB2016.docx (File 2: a copy of the data collection forms used on the training schools)
- Capacity of the Health system in managment of MK supplementary files.docx (File 3: a copy of supplementary file data showing the picture that was used for the quiz as well as additional tables showing more detail of the results)

Data are available under the terms of the Creative Commons Zero "No rights reserved" data waiver (CC0 1.0 Public domain dedication).
