# Supplementary material for: The management of microbial keratitis within Uganda’s primary health system: a situational analysis
Source: Wellcome Open Res. 2019 Sep 24;4:141. [Version 1] doi: 10.12688/wellcomeopenres.15463.1 (PMC7014927; doi:10.12688/wellcomeopenres.15463.1)
Supplement: Peer review history

# Peer review: Reviewer response for version 1

**DOI:** 10.21956/wellcomeopenres.16910.r37627

**Published:** 2020-02-25

**Authors:** Reeta Gurung

**Affiliations:** 1 Tilganga Institute of Ophthalmology, Kathmandu, Nepal

1. It would be better to include the students from the teaching institutes in the group to be the subjects to interview so that the bias from the institutes will be minimized.
2. The job descriptions of the workers of the health institution should also be looked at so that the authority who hired them are aware whether the health workers actually need to treat the eye conditions or not. If found not included in the JD, in the implementation phase even the HR section of the employer has to be inducted.

It is a good article to index because in most of the developing countries this is the situation, which again points towards the integration of eye health into the general health system of the government.

I confirm that I have read this submission and believe that I have an appropriate level of expertise to confirm that it is of an acceptable scientific standard.

# Peer review: Reviewer response for version 1

**DOI:** 10.21956/wellcomeopenres.16910.r37633

**Published:** 2020-02-11

**Authors:** Nicole Carnt, Gauri Shrestha

**Affiliations:** 1 School of Optometry and Vision Science, The University of New South Wales, Sydney, NSW, Australia

This paper states that the situation of health facilities and human resources can affect the care of MK. This paper is interesting for learning a model of primary health care in the management of MK in the developing countries. However, this article needs some revision.

**Abstract:**

 Methods:

- Mid-cadre – the term needs to be defined. Medicine schools? Or did it involve allied health workers also?

Results:

- I understand this figure also includes district-level health facilities. But the aim was focused on the PHCs. Please make it clear.
- Was the microscope the one used for the scraping of the ulcer or observing microbiological culture reports? Please specify the type of microscope. State that it is a microscope for gram staining, not slit lamp.
- What is the average duration of training dedicated to the eye unit?

Conclusions:

- Please clarify the “more is needed”.

**Introduction:**

 Paragraph 1:

- As you may have multiple of diffuse, I would change to stromal cellular inflammation.

Paragraph 3:

- Prophylactic, not treatment - is this good AMR stewardship?
- Debatable, e.g., only 11-14% of contact lens wearers with MK lose vision (Stapleton *et al.*, 2008 ^1^).

Paragraph 4:

- Why? Were they given no or wrong treatment?
- Please clarify what “missed opportunities” are.

**Methods:**    

 Study design and setting:

- Which level of HC did PHC belong to?
- How many health centres were of the PHC category?

Data collection:

- One health worker per facility: is the sample adequate?

Analysis:

- Do you mean "training key and training areas"?

 

**Results:**

 Paragraph 1:

- The authors need to define “OCO”. Are they ophthalmic nurses, ophthalmic assistants, ophthalmic technicians or optometrists?

Paragraph 3:

- Was there any control group i.e. an ophthalmologist group, as 2D representations could be difficult to interpret.
- What other diagnoses? Abrasion?
- I think it should be reserved for MK. Eye infection does not necessarily indicate MK.

Paragraph 4:

- Please elaborate on it. Were they Ayurvedic or homeopathic or something else?

Last paragraph:

- Add “fluorescein” to staining.

Table 1:

- What was the staff-to-patient recommended ratio?

 

 Figure 2:

- State this is via picture identification, as it is the title is misleading.

 

**Discussion:**

 Paragraph 1:

- Are OCOs primary eye health workers? What should be the human resource for eye health care at HC II and III levels?

Paragraph 3:

- Please rephrase “wanting in basic skills including measuring of visual acuity”.
- Please, use direct sentences quoting authors instead of mentioning another study.

Paragraph 4:

- Please rephrase "it is not unexpected that we found that...".
- Replace "they" with “the rest of them” in the sentence followed by “Imply that they…”.
- Remove "us" and state the hospital.
- Change "most containing" to "most of them containing".

Paragraph 5:

- Add "including MK" after "eye care".
- Add “topical” in front of “drugs”.

Paragraph 6:

- Please clarify “training pack”.
- Change grammar - change “targeting” to "to educate".
- Delete “how to” before “locally make…”.
- Add a full stop after “corneal staining”. Delete "and a" and begin a new sentence with "Also, a".
- Please specify, what does the WHO primary eye care training manual have to offer for the training on MK?

Paragraph 7:

- Delete "also".
- Spelling – “vials”.
- “Should be available for”, replace "lower" with an alternative term in "...be availed to lower facilities"?
- Please move and merge the whole unit with paragraph 4.

Paragraph 8:

- Change grammar to a proportion of time, "although low".
- How much weightage has been dedicated to the eye unit? 

Paragraph 9:

- Not clear what you mean - is it clinical experience?

Paragraph 10:

- Be more specific with staining.
- What do critical skills indicate?

**Conclusion:**

- Please be specific with “more is needed”.

We confirm that we have read this submission and believe that we have an appropriate level of expertise to confirm that it is of an acceptable scientific standard.

## References

[1] : The incidence of contact lens-related microbial keratitis in Australia. *Ophthalmology*.2008;115(10) : 10.1016/j.ophtha.2008.04.002 1655-62 10.1016/j.ophtha.2008.04.002 18538404

# Peer review: Reviewer response for version 1

**DOI:** 10.21956/wellcomeopenres.16910.r36865

**Published:** 2019-12-06

**Authors:** Stephanie L. Watson

**Affiliations:** 1 Discipline of Ophthalmology, Save Sight Institute, Sydney Medical School, The University of Sydney, Sydney, NSW, Australia; 2 Head Corneal Unit, Sydney Eye Hospital, Sydney, NSW, Australia

The paper states microbial keratitis is still a blinding disease and efforts are needed to address this. In the developing world, models of care affect outcomes for this condition. This paper is of interest as it examines the role of appropriate care in the management of microbial keratitis. Specific comments are:

Abstract:

- “Mid-cadre” is not a term that the readership would necessarily understand, is there another term that could be used to describe this here?
- What type of microscope?

Introduction:

- Sight loss from keratitis can even result when scarring is not severe but rather when it causes irregular astigmatism.
- Clarify the link between microbial keratitis, corneal abrasion and corneal ulcer.

 

Methods:

- Please clarify if the tertiary referral centres, are tertiary referrals for ophthalmology or for general care.
- How representative is South Western Uganda of Uganda as a whole? Can the results be generalised to Uganda?
- Is the village level a HC I and therefore is HC II the second level?
- Are the different HC levels in order of increasing complexity?
- When it is stated that “all the health facilities within the sampled districts were enrolled” does this mean all HC levels I to VII?
- Please define what is meant by mid-cadre.

 

Data collection:

- Were the questionnaires specifically designed for the study?

 

Results:

- Are district hospitals HCVII?
- Did any of the facilities have slit lamps? Cobalt blue light?
- Table 1: please describe what “staffing levels” means.

 

Discussion:

- Use either microbial keratitis or the abbreviation MK consistently.
- Please elaborate further on why steroids should not be given, this will be a good educational point for the article.
- Spelling “vails”, please correct to “vials”.
- Please comment that gram positives are typically more common causes of keratitis compared to gram negative bacteria particularly in non-contact lens wearers.

I confirm that I have read this submission and believe that I have an appropriate level of expertise to confirm that it is of an acceptable scientific standard.
